# Frequency of common *HFE *variants in the Saudi population: a high throughput molecular beacon-based study

**DOI:** 10.1186/1471-2350-7-43

**Published:** 2006-05-03

**Authors:** Osama A Alsmadi, Fadi Al-Kayal, Mohamed Al-Hamed, Brian F Meyer

**Affiliations:** 1 Arabian Diagnostic Laboratory, Research Centre, King Faisal Specialist Hospital and Research Centre, Riyadh, Saudi Arabia

## Abstract

**Background:**

Hereditary Hemochromatosis (HH) is an autosomal recessive disorder highlighted byiron-overload. Two popular mutations in *HFE*, p.C282Y and p.H63D, have been discovered and found to associate with HH in different ethnic backgrounds. p.C282Y and p.H63D diagnosis is usually made byrestriction enzyme analysis. However, the use of this technique is largelylimited to research laboratories because they are relativelyexpensive, time-consuming, and difficult to transform into a high throughput format.

**Methods:**

Single nucleotide variations in target DNA sequences can be readily identified using molecular beacon fluorescent probes. These are quenched probes with loop and hairpin structure, and they become fluorescent upon specific target recognition. We developed high throughput homogeneous real-time PCR assays using molecular beacon technology, to genotype p.C282Y and p.H63D variants. Representative samples of different genotypes for these variants were assayed by restriction enzyme analysis and direct sequencing as bench mark methods for comparison with the newly developed molecular beacon-based real-time PCR assay.

**Results:**

Complete concordance was achieved by all three assay formats. Homozygotes (mutant and wildtype) and heterozygotes were readily differentiated by the allele specific molecular beacons as reported by the associated fluorophore in the real-time assay developed in this study. Additionally, these assays were used in a high throughput format to establish the allele frequency of C282Y and H63D in Saudis for the first time.

**Conclusion:**

These assays may be reliably applied as a diagnostic test or large scale method for population screening.

## Background

Hereditary Hemochromatosis (HH) is an autosomal recessive disorder highlighted byiron-overload [1]. HH was recognized more than a century ago as a condition with three symptoms: diabetes, skin bronzing, and cirrhosis [2]. In Caucasians, HH affects 3–8 in every 1000 individuals, with an approximately 10% prevalence of heterozygous carriers [3]. The molecular basis for HH was completely unknown untilthe identification in 1996 of a gene onchromosome 6p, designated *HLA-H*, subsequently renamed *HFE *[4]. A cysteine-to-tyrosine substitution at position282 in the HFE protein (p.C282Y), has been observed in the majorityof HH patients, with frequencies ranging from 64% to 100% in subjects (fromdifferent geographic areas) who have hemochromatosis [5-7]. A substitution of histidine by aspartic acid at position 63 (p.H63D) in the HFE protein, was also linked to an additional 1–10% of HH cases [5]. Large population-based studies are required to definitively establish the prevalence of these mutations on an ethnic and or geographic basis. A recently described third mutation, which substitutes cysteine for serine at position65 (p.S65C) of the HFE protein, is enriched in HH individuals who have a mild form of the disease and lack the p.C282Y or p.H63D mutations [8]. The overall impact of this third mutation is currently unclear. Whilst many other mutations of *HFE *associated with the hemochromatosis phenotype have been described, they appear to be rare or "private" mutations [9].

The diagnosis of hemochromatosis was previously based on a combined clinical and laboratory assessment including physical examination, elevated transferrin saturation, and serum ferritin. Pedigree studies have boosted the acceptance of HH genetic screening in several countries [10]. The most established screening test for HH is transferrin saturation. The sensitivity of transferrin saturation for the diagnosis of HH has been reported to be 94% based on a population screening study from Busselton, Australia [11]. Today, the diagnosis of HH has shifted from clinical symptoms and biochemical assessment towards genetic testing. Genetic testing for *HFE *mutations now plays a central role in confirming the clinical diagnosis of HH. Several molecular methods have been used to screen for the common HH mutations, p.C282Y and p.H63D. The methods include allele-specific oligonucleotide hybridization (ASO) [12], restriction enzyme (RE) analysis [4], and the invader assay [13]. High throughput ASO methods require up to two days to complete, include multiple post PCR steps and often use radioactive reagents, all factors likely to discourage application of this format. RE also requires several post PCR steps including an overnight digestion and analysis of ethidium bromide stained gels, and can potentially lead to PCR contamination as a result of excess handling. The invader assay on the other hand, is an isothermal high throughput assay, but requires complicated design of both signal and invader probes which must work in tandem and involve multi-step intermolecular events. Genotypes are assigned after determination of the net wild-type/variant signal ratios, which can potentially lead to genotyping miscalls. The use of these techniques have enabled several *HFE *population studies, however, these techniques remainlimited to research laboratories as they are relativelyexpensive, time-consuming, and in some cases difficult to transform into a robust high throughput format.

Molecular beacons are single-stranded oligonucleotide probes with a stem-and-loop hairpin structure [14]. The loop domain of the molecular beacon is complementary to the target sequence and contains the mutation site usually embedded centrally. The molecular beacon is labeled with a flourophore at one end and a quencher, 4'-(4-dimethylaminophenylazo) benzoic acid (DABCYL), at the other end. When the specific target is encountered, the molecular beacon forms a hybrid with the target leading to conformational changes that position the flourophore away from the quencher. As a result, fluorescence of the molecular beacon is restored signaling presence of the associated allele. Under optimal conditions, instability of mismatched hybrids increases the specificity of molecular beacons, making them the perfect probes for single nucleotide polymorphism (SNP) genotyping or for detection of point/small mutations. Molecular beacons can each be conjugated to different fluorophores enabling simultaneous detection of any genotype in a closed tube homogeneous assay [14-16]. In addition, they eliminate the need for agarose gel analysis of PCR products because the entire assay fromamplification to detection is performed in the same microwell, allow SNPs to be analyzedin less than 2 hours and genotyping calls can be reliably automated.

In this report we describe the design and validation of 2 molecular beacon-based real-time PCR assays for the detection of the common HH mutations, p.C282Y and p.H63D.

## Methods

### DNA samples

A total of 23 samples of known genotypes for the p.C282Y and p.H63D mutations were selected and anonymized prior to being used in assay development and validation. The samples originated from patients referred to our laboratory for *HFE *genotyping. Routinely, these samples are assayed using a restriction digestion based methodology. For population studies, we utilized a total of 540 random, anonymized newborn dried blood spots (DBS) as a source of template DNA. The neonatal DBS are routinely sent from multiple birth centers across Saudi Arabia to the National Laboratory for Newborn Screening in our institution. The likelihood of these samples originating from closely related subjects is virtually negligible but cannot be completely excluded. Sample collection and enrollment in this study was approved by our institutional review board (IRB) according to guidelines of the Declaration of Helsinki.

### Whole genome amplification (WGA)

DNA was amplified from dried blood spots using a modified WGA method [17]. A Repli_g 100 kit (Qiagen, Valencia, CA) was used for amplification. In brief, 1.2 mm punch biopsies were derived from the dried blood spots using a Harris Micro Punch (Whatman Inc, USA), and dispensed into 96-well plates. The punch biopsies were then soaked with 20 μl of PBS, followed by addition of 20 μl of lysis solution A according to the manufacturers recommendations. The plates were allowed to sit on ice for 10 minutes to allow release of nuclei from the discs. Each sample was then neutralized by adding 20 μl of solution B. From each lysis mix, 2 μl was transferred to 8 μl of the WGA reaction mix present in each well of a 96-well micro plate, bringing the final reaction volume to 10 μl per sample. Reaction mixtures were incubated for 16 hr at 30°C on an MJ Research thermocycler (MJ Research, Watertown, MA, USA)and terminated by heating to 65°C for 5 min. Dilutions from the crude amplification mixture were directly used for genotyping.

### *HFE *genotyping by restriction enzyme digestion

Restriction digestion was carried out as described [4]. Briefly, two amplicons encompassing the p.C282Y and p.H63D mutation sites were generated by PCR prior to restriction digestion analysis. Amplification was performed in total volume of 25 μl. PCR reactions were carried out using an MJ Research thermocycler (MJ Research, Watertown, MA, USA). Each PCR reaction contained 100 ng gDNA, 1X PCR buffer, 1.5 mM MgCl_2_, 0.25 mM dNTPs, 0.5 μM forward/reverse primers, and 1U *Taq *polymerase (Qiagen, Valencia, CA). The primers TGGCAAGGGTAAACAGATCC and CTCAGCTCCTGGCTCTCAT were used to generate a 357 bp genomic fragment that contained the c.845G>A mutation site in the *HFE *gene. Primers ACATGGTTAAGGCCTGTTGC and CTTGCTGTGGTTGTGATTTTCC were used to generate a 294 bp genomic fragment that contains the c.187C>G mutation site in the *HFE *gene. In each instance, amplification was performed after an initial activation step for *Taq *polymerase of 15 minutes at 95°C followed by 35 cycles each of 1 min at 95°C, 1 min at 56°C, and 1 min at 72°C. A final extension step of 10 min at 72°C was also used. Following PCR, restriction enzyme digestion reactions were set up as follows: 10 μl PCR product, 2 μl restriction enzyme (RE), and 1X RE buffer. *RsaI *(10 u/μl), and *MboI *(10 u/μl) (Stratagene, La Jolla, CA, USA), were used to digest the c.845G>A and c.187C>G mutation-containing fragments respectively. Digestion products were run on a 2% agarose gel for 30 min at 120V.

### PCR and direct sequencing

Amplicons encompassing the p.C282Y and p.H63D mutation sites of HFE were generated by PCR amplification using gDNA samples. Primer pairs used generated 388 bp and 486 bp fragments for p.C282Y and p.H63D respectively. Sequencing reactions were performed using an Amersham ET Dye Terminator sequencing kit (Amersham Biosciences, Piscataway, NJ, USA) following the manufacturers instructions. Sequencing reactions were desalted and unincorporated nucleotides removed using ethanol precipitation, and then re-suspended in a formamide EDTA solution for injection on a MegaBACE 1000 capillary electrophoresis system (Molecular Dynamics, Sunnyvale, CA, USA). Sequence analysis was performed using the SeqMan module of the Lasergene software package (DNA Star Inc, Madison, WI, USA) by comparing alignments of multiple samples with each other and a Genbank reference sequence (U60319).

### Genotyping by molecular beacon assays

Real-time small scale PCR assays were performed in 0.2 ml clear flat top tubes (Phenix, Hayward, CA, USA) for use with the Rotor Gene 3000 (Corbett Research, Sydney, Australia). For large scale assays, 384-well clear optical reaction plates were used with the ABI Prism 7900 HT sequence detection system (Applied Biosystems, Foster City, CA). PCR mixtures consisted of 1X qPCR mastermix plus (Eurogentec, Seraing, Belgium), 0.5 μM forward/reverse primers, 0.25 μM of each molecular beacon probe, and 20 ng of gDNA template. Reaction tubes were subjected to an initial 2 min incubation at 50°C (Uracil-N-glycosylase will eliminate carry over amplicon contaminants in this step), then a 10 min incubation at 95°C for *Taq *polymerase activation, followed by 40 PCR cycles. Cycling conditions were 95°C denaturation for 10 sec, 58°C annealing for 30 sec, and 72°C extension for 30 sec. A negative control (deionised water) was included in each assay. The following allele-specific molecular beacons and primers were designed and used in the assays (all were sourced from Proligo, Paris, France):

#### p.C282Y assay

Wild type beacon: FAM-ccggc AGATATACGT**G**CCAGGTGG gccgg-DABCYL

Mutant beacon: TET-ccgcc GAGATATACGT**A**CCAGGTGGA ggcgg-DABCYL

Forward primer: GGCTGGATAACCTTGGCTGT

Reverse primer: GATCACAATGAGGGGCTGAT

#### p.H63D assay

Wild type beacon: FAM-ccgctg CTATGAT**C**ATGAGAGTCGCC cagcgg-DABCYL

Mutant beacon: TET-ccgctg CTATGAT**G**ATGAGAGTCGCC cagcgg-DABCYL

Forward primers: CTTTGGGCTACGTGGATGAC

Reverse primer: TGGCTTGAAATTCTACTGGAAA

In the beacon sequences, underlined lower case bases refer to the complementary arms. Bolded and capitalized bases represent the allelic variants at each locus. For the beacons design, we have used mfold [18] for appropriate probe folding. Genotype calling of DNA samples was determined by measuring the threshold cycle (Ct) value for each sample. Fluorescence data was collected during each annealing step throughout cycling at which point the molecular beacon is bound to its complementary target amplicon. Results were displayed as an amplification plot for fluorescence versus cycle number. The threshold cycle is the cycle at which the fluorescent signal is first reported above the set baseline fluorescence. The baseline fluorescence was set at 10% above the average fluorescence value of PCR cycles 1 through 18. Genotyping of DNA samples was based on positive threshold cycles for FAM, TET, or both. Positive Ct values were usually detected between cycles 25–30, and indicated the presence of specific allele(s).

## Results

### C282Y and H63D genotyping of *HFE *by PCR/restriction enzyme analysis

**Figure 1:**
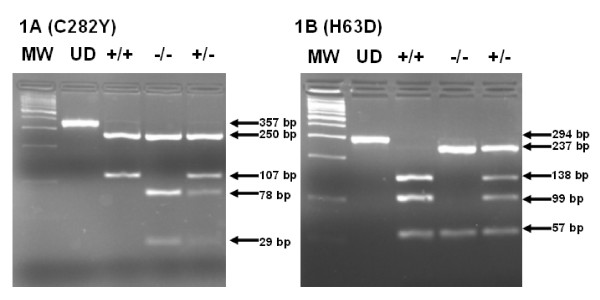
2% agarose gel showing the restriction digestion fragments for detection of p.C282Y (1A) and p.H63D (1B) mutations. Undigested (UD), normal (+/+), mutant (-/-), and heterozygote (+/-).

Both of p.C282Y and p.H63D mutations alter restriction-enzyme sites, providing a method for genotyping. c.845G>A creates a recognition site for *RsaI*; c.187C>G abolishes the DNA sequence recognition site for *MboI. RsaI *was used for restriction analysis of an amplicon (357 bp undigested; Figure 1A, lane 2) containing the p.C282Y mutation site of *HFE*. Representative restriction analysis using the control samples is shown in Figure 1. Following *RsaI *digestion a homozygous wild type sample (GG) produces two fragments (Figure 1A, lane 3: 250 and 107 bp), whereas a homozygous mutant sample (AA) results in three fragments (Figure 1A, lane 4: 250, 78, and 29 bp). For heterozygotes, four fragments (Figure 1A, lane 5: 250, 107, 78, and 29 bp) were present following *RsaI *digestions. Similarly *MboI *was used for restriction analysis of an amplicon (294 bp undigested; Figure 1B, lane 2) containing the p.H63D mutation site of *HFE*. Following *MboI *digestion a homozygous wildtype sample (CC) produces three fragments (Figure 1B, lane 3: 138, 99 and 57 bp), whereas a homozygous mutant sample (GG) generates two fragments (Figure 1B, lane 4: 237 and 57 bp). For heterozygote sample (CG) four fragments (Figure 1B, lane 5: 237, 138, 99, and 57 bp) were present following digestion.

### Direct sequencing of *HFE *amplicons containing the p.C282Y and p.H63D sites

Direct sequencing of amplicons containing the p.C282Y and p.H63D sites of *HFE *were performed on both strands for all 23 patients and three control samples. Sequencing results of all 23 samples and the controls were 100% concordant with that achieved using PCR and restriction analysis.

### p.C282Y and p.H63D genotyping by molecular-beacon based real-time PCR assays

**Figure 2:**
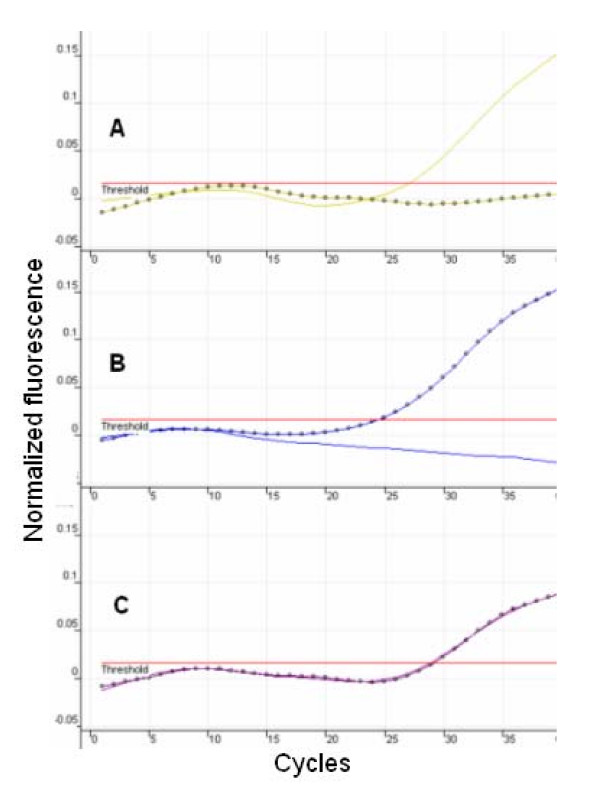
FAM signal;  TET signal  Representative real-time PCR genotyping results for p.C282Y variants are shown. (A) Normal (FAM positive, TET negative), (B) mutant genotype (FAM negative TET positive), and (C) heterozygote (FAM positive TET positive).

**Figure 3:**
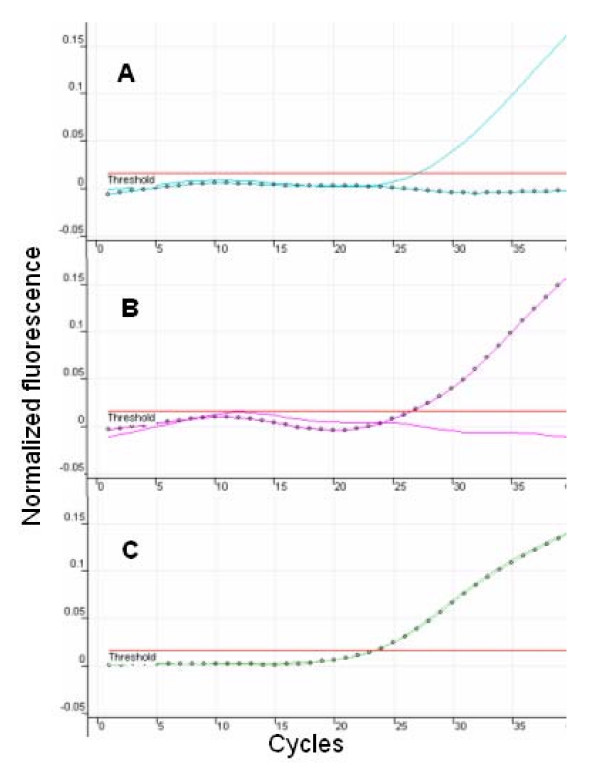
FAM signal;  TET signal  Representative real-time PCR genotyping results for p.C282Y variants are shown. (A) Normal (FAM positive, TET negative), (B) mutant genotype (FAM negative TET positive), and (C) heterozygote (FAM positive TET positive).

p.C282Y and p.H63D genotyping was carried out by using two independent molecular beacon-based homogeneous assays. Representative results of assays for C282Y and p.H63D using sequence validated controls are shown in Figures 2 and 3 respectively. These results were generated using the Corbett 3000 system; identical profiles are also observed when the assay is run on the ABI Prism 7900 HT sequence detection system. Ct values were detected in these assays usually between cycles 25 and 30, and indicate the presence of specific allele(s) depending on the positive signal(s) that are detected. For both of these assays, wild type and variant allele-detecting molecular beacons were labeled with FAM and TET respectively. FAM channel only positive signal indicated a homozygous wild type genotype. Conversely TET channel only positive signal indicated a homozygous mutant genotype. FAM positive/TET positive channels indicate a heterozygote genotype. p.C282Y and p.H63D real-time assay cycling parameters were optimized to be identical thus permitting simultaneous analysis of both genotypes, albeit in independent reactions. Genotyping of p.C282Y and p.H63D variants was accomplished by real-time PCR amplification of C282Y- and H63D-containg amplicons in the presence of the allele-specific molecular beacons for these variants. Relative fluorescence was monitored using the Rotor Gene 3000 real-time thermal cycler, and representative genotyping results are shown in Figures 2 (p.C282Y) and 3 (p.H63D). Using this real-time PCR method, genotyping of all 23 test samples was in complete concordance with the results achieved using restriction enzyme analysis and direct sequencing.

### High throughput population genotyping for p.C282Y and p.H63D

Here we analyzed a total of 540 DNA samples obtained from anonymized newborn dry bloodspots. DNA was amplified from these spots using whole genome amplification (WGA) technology. WGA provides a comprehensive whole genome coverage and minimal loci bias [19]. The WGA method provides unlimited quantities of DNA directly from a variety of samples types including dried blood spots. p.C282Y and p.H63D genotyping was facilitated by the use of the two molecular beacon assays which we developed and that were readily applicable for genotyping of a large number of samples. Using this approach on a cost per sample basis in respect of both reagents and time the molecular beacon based assay was more economical than restriction enzyme analysis or direct sequencing. This is based upon a requirement for 1,000 or more assays which introduces an economy of scale with respect to the synthesis of molecular beacons and assumes availability of real-time PCR instrumentation. Based upon these numbers, the reagent cost of each of the beacon assays described here. has been calculated at <US$ 1.0, This is compared to a 3–4 fold more reagent cost when alternative methodology such as RE or sequencing is considered and reflects the single step beacon procedure as opposed to the multi-step nature of alternatives. Clearly the single step beacon procedure is similarly advantageous when viewed in terms of technician time. The overall p.C282Y genotyping resulted in no mutant alleles being detected as all subjects were homozygous (GG) for the wildtype allele. This result indicates the mutant or variant A allele to be very rare (frequency < 0.001) in the Saudi population. On the other hand, p.H63D genotyping analysis showed genotypes as follows: 380 (70.4%) subjects were wildtype homozygotes (CC), 129 (23.9%) subjects were heterozygotes (CG) and 31 (5.7%) were homozygote mutants (GG). Based on these genotypes, the Saudi allele frequencies are 0.823 and 0.177 for the normal (C) and risk (G) alleles respectively at this locus. Based on the risk allele frequencies observed in this study, C282Y mutation (< 0.001) was comparable to that reported in Bulgaria and lower than all other population studies published thus far. The H63D (0.177) was higher than most of the European populations, Hispanics, Asians, but less than that observed in Bulgarians, Spanish, and Portuguese [20].

## Discussion

Three genotyping methods were compared in this study independently using DNA samples representing all possible genotypes of the p.C282Y and p.H63D mutations of HFE. We have developed and validated two new molecular beacon-based real-time PCR genotyping assays, and performed a head to head comparison against the restriction enzyme digestion and direct sequencing reference methods. Even though *HFE *genotyping in other laboratories is already automated and does not provide a major technical obstacle, molecular beacon-based real-time PCR genotyping may provide a cheaper and more efficient alternative, particularly for large studies.

Both the new real-time PCR assays provided unambiguous genotyping in total concordance with reference methods. These real-time assays were simple, inexpensive, fast, and less labor intensive in relation to restriction digestion-based mutation analysesand direct sequencing. Therefore, the closed-tube allelic discrimination assays developed by this study have several advantages over other mutation detection techniques: a) the hairpin-shaped molecular beacons are extremely specific in distinguishing single base-pair variants; b) there is significant reduction of post-PCR cross-contamination risk, and c) the technology can readily be adapted to a high-throughput format (e.g., 96 or 384-well assays). For this purpose, we used the molecular beacon based assay for analysis of 540 random anonymized DNA samples derived from Saudi newborn subjects, using the 384-well genotyping format and the ABI Prism 7900 HT sequence detection system. Genotyping could be performed in less than two hours per run allowing the analysis of thousands of DNA samples per day. Such a true high throughput setting is essential to the performance of large population-based studies for the establishment of allele frequencies. The establishment of population based allele frequencies for common disease related mutations or variants are a necessary prelude to the clinical use of genotyping of these loci.

Population *HFE *studies for most of the world regions are missing, and previous genotyping studies indicated that siblings of non-expressing (asymptomatic) homozygotes have been found to have iron overload [10,21]. In the Saudi population incidence of the *HFE *A (or risk allele of P.C282Y), which is the primary mutation underlying HH in other populations, was extremely low (<0.001). Alternately, incidence (0.177) of the C or risk allele underlying the *HFE *p.H63D mutation was relatively common based upon reports in other populations [4,22,23]. Collectively the data suggests that the Saudi population is either at low risk for HH or that novel mutations of HFE may play a role in this population. The high incidence of p.H63D would elevate risk but does not have as strong an association with HH as p.C282Y [11,24-26]. Sequencing of *HFE *in clinically well characterized patients of Saudi origin with HH is indicated to clearly establish the role of p.H63D or novel *HFE *mutations in this population.

## Conclusion

The two assays which we have developed and presented here possess adequate target specificity and are readily performed in a high throughput format. These assays may be reliably applied as a diagnostic test or large scale method to facilitate world-wide population based studies.

## Abbreviations

Hereditary Hemochromatosis (HH)

Single Nucleotide Polymorphism (SNP)

Institutional Review Board (IRB)

Allele-specific oligonucleotide (ASO)

Restriction Enzyme (RE)

Whole Genome Amplification (WGA)

Dry Blood Spots (DBS)

## Competing interests

The author(s) declare that they have no competing interests.

## Authors' contributions

OAA drafted the manuscript, designed and coordinated the study, and analyzed the data. FA carried out the molecular genetic studies and analyzed the data. MA assisted in carrying out the molecular genetic studies. BFM edited and contributed to the manuscript, and co-designed the study.

## Pre-publication history

The pre-publication history for this paper can be accessed here:


